# Mathematical Physics Analysis of Nozzle Shaping at the Gas Outlet from the Aperture to the Differentially Pumped Chamber in Environmental Scanning Electron Microscopy (ESEM)

**DOI:** 10.3390/s24103243

**Published:** 2024-05-20

**Authors:** Jiří Maxa, Vilém Neděla, Pavla Šabacká, Tomáš Binar

**Affiliations:** 1 Institute of Scientific Instruments of the CAS, Královopolská 147, 612 64 Brno, Czech Republic; 2 Faculty of Electrical Engineering and Communication, Brno University of Technology, Technická 10, 616 00 Brno, Czech Republic

**Keywords:** Ansys Fluent, ESEM, critical flow, nozzle, CFD, numerical simulation

## Abstract

A combination of experimental measurement preparations using pressure and temperature sensors in conjunction with the theory of one-dimensional isentropic flow and mathematical physics analyses is presented as a tool for analysis in this paper. Furthermore, the subsequent development of a nozzle for use in environmental electron microscopy between the specimen chamber and the differentially pumped chamber is described. Based on experimental measurements, an analysis of the impact of the nozzle shaping located behind the aperture on the character of the supersonic flow and the resulting dispersion of the electron beam passing through the differential pumped chamber is carried out on the determined pressure ratio using a combination of theory and mathematical physics analyses. The results show that nozzle shapes causing under-expanded gas outflow from the aperture to the nozzle have a worse impact on the dispersion of the primary electron beam. This is due to the flow velocity control. The controlled reduction in the static pressure curve on the primary electron beam path thus causes a significantly higher course of electron dispersion values than variants with shapes causing over-expanded gas outflow.

## 1. Introduction

This paper deals with the issue of the vacuum chamber pumping of an environmental scanning electron microscope (ESEM). Its content builds on and expands the field of research from previously published articles [1,2]. In this paper, the issue of the differentially pumped chamber pumping and the nature of the flow behind the aperture separating the specimen chamber from the differential pumped chamber is solved, which is of key importance for the primary electron beam dispersion that passes through the medium.

**Figure 1:**
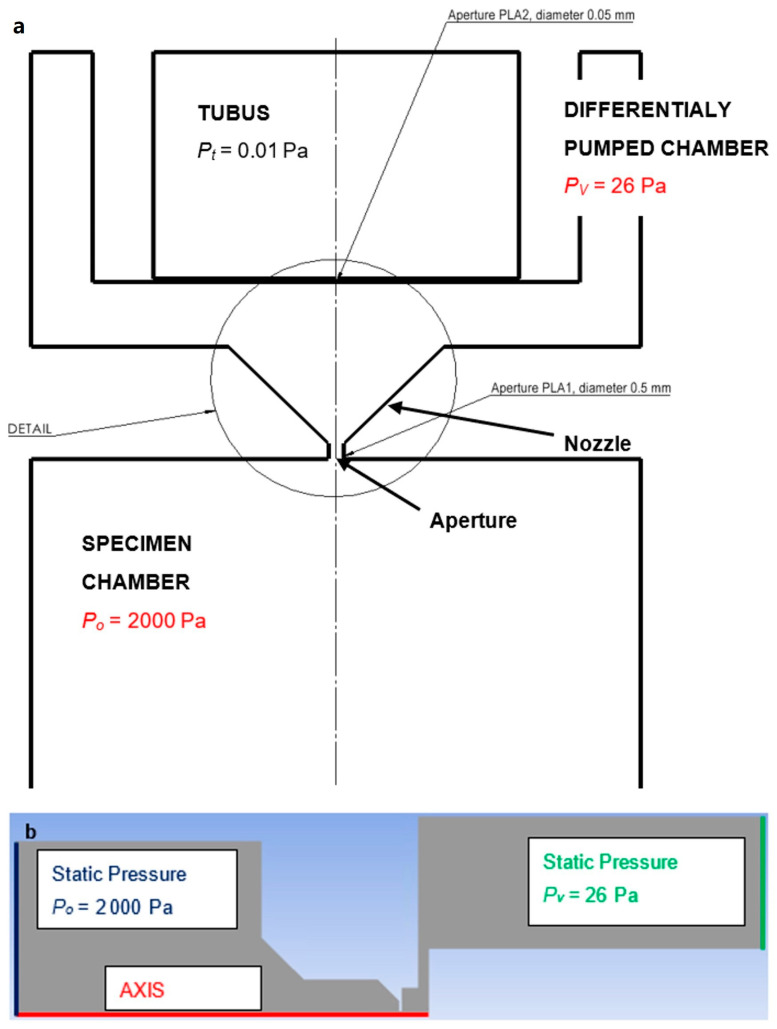
Environmental scanning electron microscope (ESEM)—chamber diagram (**a**), 2D axisymmetric model with boundary conditions (**b**).

Electron microscopy has brought the possibility of observation without the need for a light source and at zooms that are several times more detailed than conventional optical microscopes. The environmental scanning electron microscope (ESEM) has been developed without the need for a vacuum [3]. In the ESEM, it is possible to observe electrically nonconductive, semiconducting [4,5], or native samples [6] without damaging them or to study these samples in dynamic in situ experiments [7]. Signal electrons are detected in the ESEM by special ionization or scintillation detectors. The ESEM differs from the conventional type of electron microscope by the addition of a differentially pumped chamber. This chamber separates the specimen chamber from the tube. Due to the large pressure difference between the tube (0.01 Pa) and the specimen chamber (usually around 600–2000 Pa), the two chambers cannot be separated by just one opening (aperture). The reason is that it would not be possible to achieve such a low pressure in the tube by pumping [8,9,10]. Therefore, a differentially pumped chamber is inserted into this intermediate space in the ESEM. It can separate these pressure differences by separating the tube and specimen chamber. Each chamber is, thus, separated by a small aperture. This results in pressure losses between the differentially pumped chamber and the tube of approximately 80 Pa to 0.01 Pa, and subsequently between the specimen chamber and the differential pumping chamber in the ratio of approximately 2000 Pa to 80 Pa. This state is schematically illustrated in Figure 1a. Figure 1b shows the 2D axisymmetric model with boundary conditions for the analyses described below. The method of boundary condition selection will be given later in this article.

**Figure 2:**
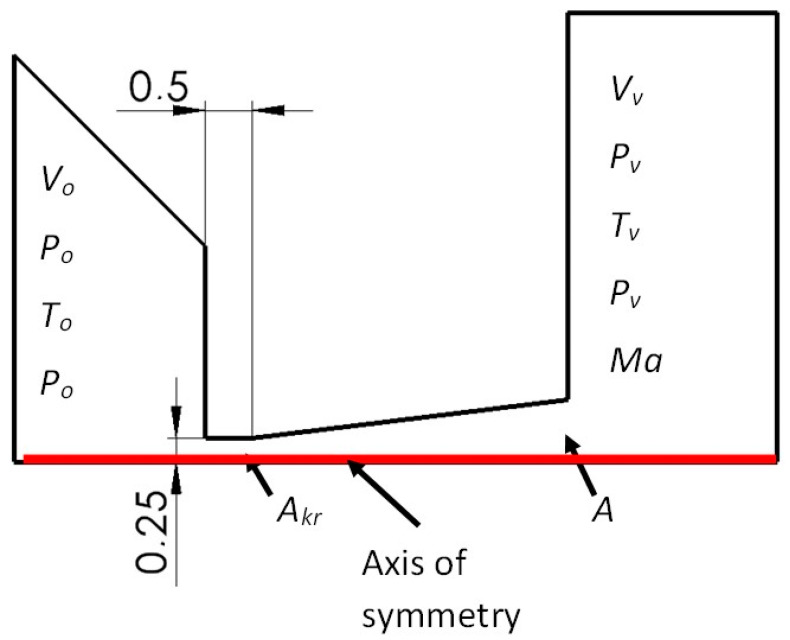
Two-dimensional axisymmetric DETAIL area rotated by 90°.

Later in this article, the area marked as DETAIL in Figure 1 will be solved. This area is then plotted and shown in Figure 2. In this figure, this DETAIL is modified for mathematical physics analyses as a 2D axisymmetric shape rotated by 90°. Analyses are then performed in the aperture and nozzle area in a differentially pumped chamber. The analyses are based on the predetermined dimensions used in the ESEM. This is an aperture diameter of 0.5 mm, an aperture length of 0.5 mm, and a distance between PLA 1 and PLA 2 of 5.5 mm.

High demands are placed on the shaping of this aperture fitted with a nozzle. The role of this design is to maintain the pressure gradient between the two chambers. Another role of this design is to model the gas flow so that the courses of static pressure and its gradients, or the resulting shock waves, are of such a nature that they cause the least possible number of iterations of gas molecules with passing electrons. Each interaction of a gas molecule with an electron causes a deflection from its path and reduces the resulting sharpness of the image.

The main contribution of this paper is the research on the shaping of the space above the aperture in the conditions of the ESEM type AQUASEM II. This microscope was built at the Institute of Scientific Instruments of the Czech Academy of Sciences by a team led by Vilém Neděla.

This paper also deals with the issue of experimental pressure sensing in chambers to obtain input data for numerical modeling. Dr. Danilatos has been dealing with this issue for a long time [3,11,12]. It is possible to cite several publications from which it was drawn.

In the article gas-flow field in the environmental sem, the author wrote that a conically shaped PLA is preferred for several reasons; one such reason is that it allows the preservation of a stagnant pressure environment over the specimen surface for a short distance down to one PLA diameter. From the number density contours, no effect is observed over the specimen surface. The same observation has been made from speed, temperature, and surface-pressure determinations [13]. His next work established that a thin-plate PLA assembly produces the minimum electron beam current loss at the high-vacuum–high-pressure boundary. This was expected because the thin PLA walls result in the most abrupt change in pressure, which generates the fastest gas density decrease along the axis. The thicker PLA design creates a slower pressure transition, which leads to a greater loss in the beam current above the PLA [14]. These considerations have been developed by Dr. Danilatos in his other works.

In the article electron beam loss in commercial esem [15], the author generally deals with the issue of when an electron beam in an ESEM suffers small but significant losses in the differentially pumped chamber before it enters the specimen chamber. It also describes the losses caused by the flowing gas behind the aperture. These increase with the pressure in the specimen chamber and eventually become disastrous. The author proves that these effects are minimal in an optimal construction system.

In the article beam transfer characteristics of a commercial environmental sem and a low vacuum sem [16], it is possible to become acquainted with a basic study of density and pressure curves of gas flow through a small opening separating two chambers with a large pressure gradient. In this article, Dr. Danilatos uses the example of an FEI electron microscope to analyze the gas flow through an aperture as a function of its size and controlled back pressure. Furthermore, Dr. Danilatos, in his publication figure of merit for environmental sem and its implications [17], discusses the size of the angle and thickness of the nozzle without an opening. Finally, it adds the advantage of a larger nozzle opening and contradictory conditions in terms of nozzle thickness. For optimal passage of the primary electron beam, the thinnest nozzle possible is suitable. On the other hand, for the ability of vacuum pumps to achieve a sufficient pressure drop between chambers separated by an aperture, a nozzle as thick as possible is suitable.

In another publication, optimum beam transfer in the environmental scanning electron microscope [18], he also deals with the optimization of the electron beam passage depending on the density distribution along this path. He points out that the thin nozzle represents the minimum thickness of the increased pressure that the electron beam must overcome during its transmission.

The above-mentioned analyses in our paper are followed by a study of the flow nature and its impact on the passage of the primary electron beam in AQUASEM II. An analysis of the impact of the selected shapes of the nozzle over the aperture on the impact of the primary electron beam dispersion for the dimensions of the space and aperture diameter and the experimentally obtained pressure ratio was performed [19,20].

These analyses were used as a basis for the construction of the differentially pumped chamber shape in the area of the primary electron beam passage. These analyses, together with the experiments carried out in the experimental chamber, simulate the work of a given part of the microscope.

These analyses are part of comprehensively performed experiments for supersonic flow mapping and its impact on the primary electron beam dispersion, as will be presented in this paper. Supersonic flow occurs when pumping a differentially pumped chamber in the ESEM [21,22]. This research will be used for the subsequent design of a differentially pumped chamber of next-generation microscopes [23,24].

## 2. Methodology

### 2.1. Experimental Measuring

In the beginning, experimental sensing of pressure conditions between the specimen chamber and the differentially pumped chamber was performed. The sensing was performed to determine the normal operating conditions and the ability of vacuum pumps to maintain the given initial ratio at the PLA 1 operating aperture with a diameter of 0.5 mm.

Experimental measurement of the pressure distribution under operating conditions in the specimen chamber was performed using the Pfeiffer CMR 361 (Pfeiffer Vacuum Technology AG, Aßlar, Germany) pressure sensor [25]. The measurement of the pressure distribution was performed using the Pfeiffer CMR 362 (Pfeiffer Vacuum Technology AG, Aßlar, Germany) pressure sensor in the differentially pumped chamber. Both pressure sensors’ specifications are listed in Table 1.

**Figure 3:**
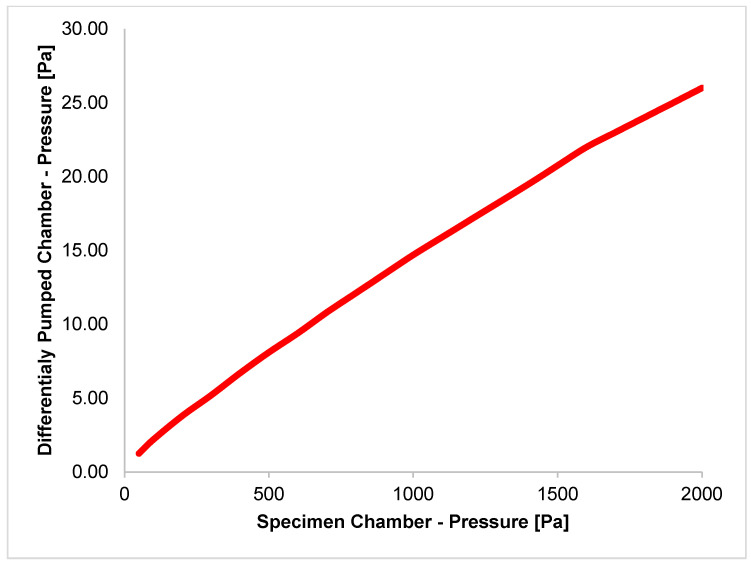
Pressure ratio between the specimen chamber and differentially pumped chamber.

Experimental measurements revealed the ability to maintain pressure conditions when using a Lavat RV 100/1 (LAVAT, a.s., Radim u Kolína, Czech Republic) rotary oil vacuum pump with a pumping speed of 0.00694 m^3^·s^−1^. The pressure conditions are shown in Table 2 and plotted in the graph in Figure 3. The pressure ratio of the pressure gradient *p*_0_ = 2000 Pa and *p_v_* = 26 Pa was chosen for subsequent analyses (marked red in Table 2) based on experimental measurements.

### 2.2. Analyzed Variants

The first chosen variant was the one with a designed Laval nozzle of a linear conical shape behind the aperture. The nozzle opening angle was such that it corresponded to the planned pressure drop between the specimen chamber and the differentially pumped chamber for the selected aperture diameter of 0.5 mm. This pressure drop, in accordance with the diameter of the aperture opening, defined the shape of the supersonic flow behind the aperture. The first variant, thus, was the calculated cross-section variant. This shape was designed using the theory of one-dimensional isentropic flow.

The nozzle design is shown in Figure 2, along with a description of the quantities for the theory of one-dimensional isentropic flow. Figure 2 shows the nozzle rotated by 90° compared to the DETAIL in Figure 1 and solved as a 2D axisymmetric calculation.

The theory of one-dimensional isentropic flow is based on the relationships that determine the ratios between pressures, densities, velocities, and Mach numbers between the area of nozzle input, in the nozzle, and within the computational cross-section of the gas expansion behind the nozzle [26,27].

For isentropic flow, the following relationships apply:

(1) $$\frac{v_{v}}{v_{kr}}={\left[\frac{\left(\varkappa +1\right)Ma^{2}}{2+\left(\varkappa -1\right)Ma^{2}}\right]}^{\frac{1}{2}}\;$$

(2) $$\frac{v_{v}}{v_{o}}={\left[\frac{2}{2+\left(\varkappa -1\right)Ma^{2}}\right]}^{\frac{1}{2}}$$

(3) $$\frac{T_{v}}{T_{o}}=\frac{2}{2+\left(\varkappa -1\right)Ma^{2}}$$

(4) $$\frac{p_{v}}{p_{o}}={\left[\frac{2}{2+\left(\varkappa -1\right)Ma^{2}}\right]}^{\frac{\varkappa }{\varkappa -1}}$$

(5) $$\frac{\rho_{v}}{\rho_{o}}={\left[\frac{2}{2+\left(\varkappa -1\right)Ma^{2}}\right]}^{\frac{1}{\varkappa -1}}$$

(6) $$\frac{\rho_{v}}{\rho_{kr}}=\frac{A_{kr}}{A}=Ma{\left[\frac{\varkappa +1}{2+\left(\varkappa -1\right)Ma^{2}}\right]}^{\frac{1}{2}\frac{\varkappa +1}{\varkappa -1}}$$

where *p*_0_ is the input pressure, *p_v_* is the output pressure, *T*_0_ is the input temperature, *T_v_* is the output temperature, *v*_0_ is the input velocity, *v_v_* is the output velocity, *v_kr_* is the critical velocity, *ρ*_0_ is the input density, *ρ_v_* is the output density, *Ma* is the Mach number, ϰ is the gas constant = 1.4, *A* is the computational cross-section, and *A_kr_* is the critical cross-section.

In the case given in this article, the ratio is *p_v_* = 2000 Pa to *p*_0_ = 26 Pa, i.e., the ratio is 0.013. The Mach number, which is 3.5 Mach, is then possible to obtain from Equation (4).

By substituting the obtained Mach number into Equation (6), it is possible to obtain the size of the output cross-section *A* at the point where the nozzle ends (Figure 1). This is possible, in our case, when the input cross-section *A_kr_* value is given by the specified aperture diameter of 0.5 mm. From a given cross-section, and thus diameter, it is possible to obtain the opening angle of the nozzle. In our case, this quantity comes out at an angle of 7° from the axis of symmetry. Other quantities are *v_v_* = 651 m·s^−1^; *ϱ_v_* = 0.00106 kg·m^−3^; *T_v_* = 86 K for *T_o_* = 297.15 K.

This is a theoretical calculation of the ideal state without the Slip Flow regime for flow in open space. Slip Flow and closed space cause flow braking and a slight change in the actual values from the calculated values, as will be seen later in the results [28]. However, the character of the flow is close to the computational state and fulfills the conditions required for us to obtain the flow character in the computational state, as will be evident from the figures of the distribution of quantities [29]. Other variants are further derived from the calculated cross-section variant.

#### 2.2.1. Calculated Cross-Section Variant

**Figure 4:**
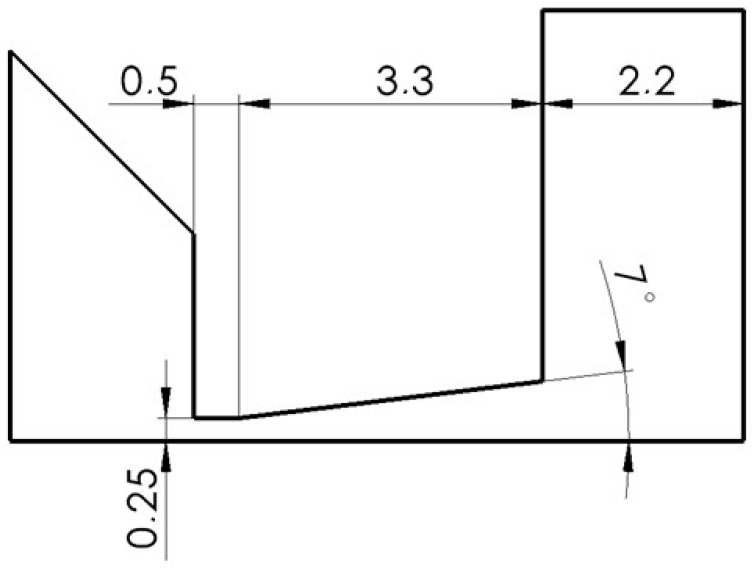
Calculated cross-section variant.

In this variant, a Laval nozzle of a linear conical shape with a so-called computational dimension was designed behind the aperture (Figure 4). This shape is based on the opening angle of 7° from the symmetry axis, which provides controlled expansion, as mentioned.

#### 2.2.2. Under-Expanded Variant

**Figure 5:**
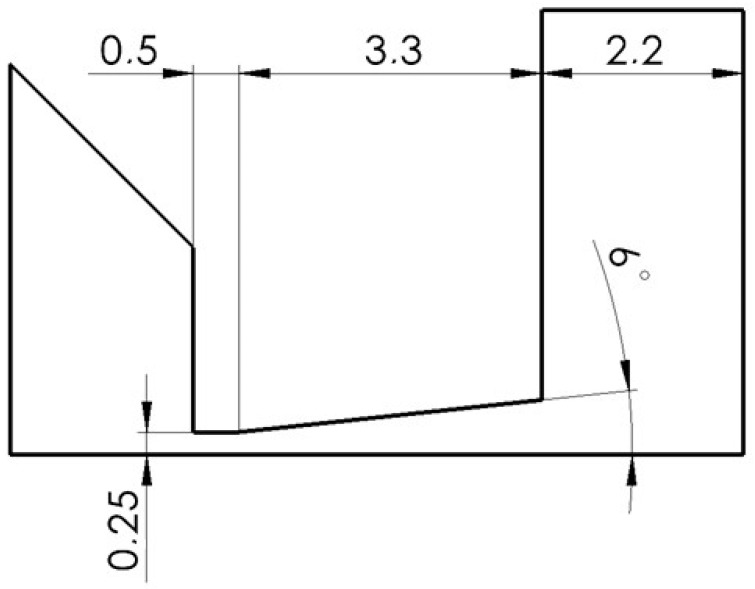
Under-expanded variant.

An angle of a smaller dimension than in the calculated cross-section variant was chosen in this variant, which causes under-expanded gas flow (Figure 5). The opening nozzle angle of 6° was therefore chosen.

#### 2.2.3. OPEN TOTAL Variant

**Figure 6:**
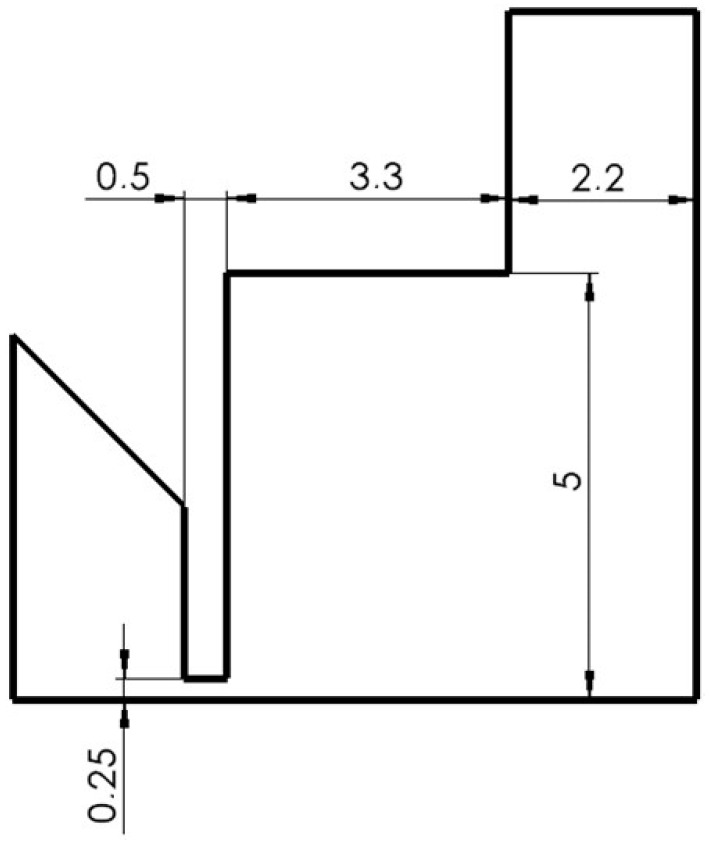
OPEN TOTAL variant.

The shape of a large opening of the area above the aperture was chosen in this variant (Figure 6). The opened area caused an uncontrolled gas expansion behind the aperture that did not affect the flow from the side.

#### 2.2.4. OPEN Variant

**Figure 7:**
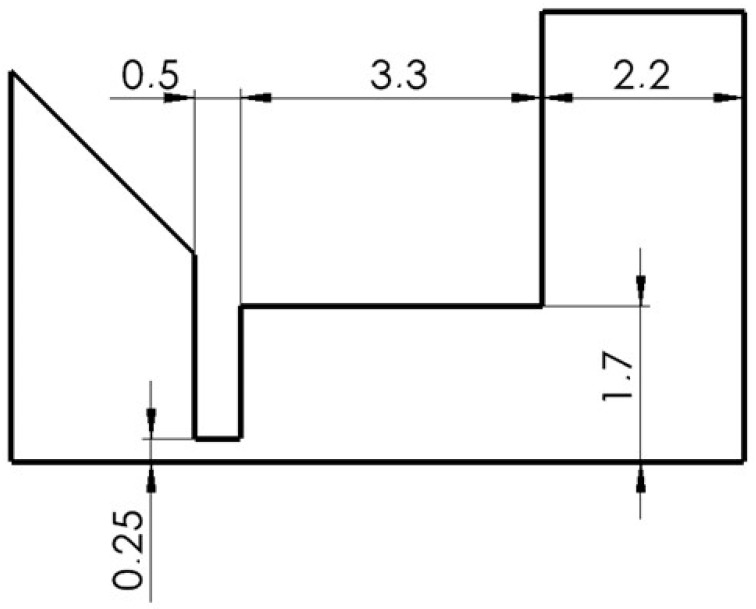
OPEN variant.

The shape of a one-third opening of the area above the aperture was chosen in this variant (Figure 7). This was to verify the effect of the partial influence of uncontrolled expansion from the side.

#### 2.2.5. Angle 45° Variant

**Figure 8:**
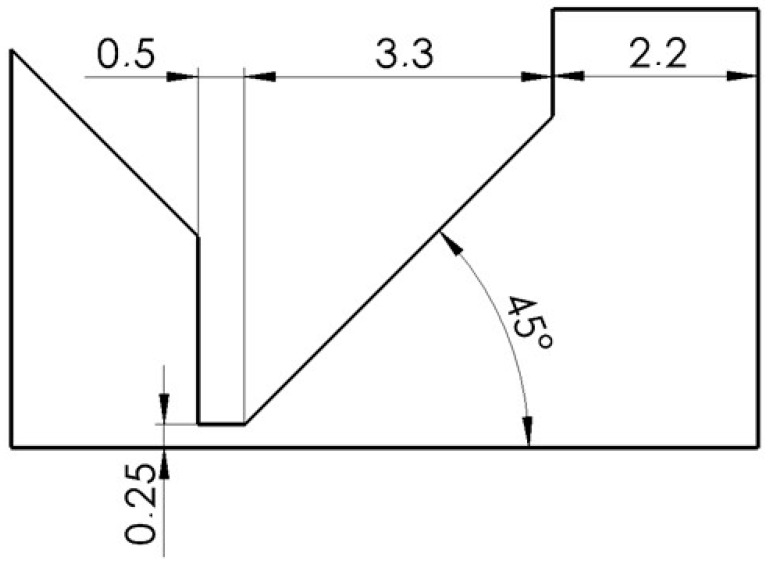
Angle 45° variant.

For another variant, the shape of the nozzle above the aperture with a large opening at an angle of 45° was chosen (Figure 8). It was a combination of a conical nozzle and an open space.

### 2.3. Tuning of Calculations in Ansys Fluent System

As a first step, the Ansys Fluent system was tuned as described in the earlier steps of this research work. This is for the analysis of supersonic critical flow on the border of continuous mechanics. The system has been tuned for use in vacuum chamber pumping research in the ESEM [30]. The results of experimental measurements were thus harmonized with mathematical physics analyses. This combination of experimental measurements and mathematical physics analyses is the greatest advantage of modern research methodology [31,32].

The experimental measurement of pressure conditions between the specimen chamber and the differentially pumped chamber was first carried out to solve the analyses described in this paper. Experimental measurements were performed using Pfeiffer CMR pressure sensors. From the range of pressure conditions, the pressure gradient *p_v_* = 2000 Pa to *p*_0_ = 26 Pa, i.e., a ratio of 0.013, was selected for subsequent analyses.

Subsequently, using the theory of one-dimensional isentropic flow, the analytical calculation dimension of the Laval nozzle was determined [33]. This dimension would provide controlled gas expansion between the specimen chamber and the differentially pumped chamber. The initial dimensions, such as the length of the nozzle and the diameter of the aperture in front of the Laval nozzle, were taken from the AQASEM II electron microscope.

From this calculated cross-section nozzle shape, under-expanded and over-expanded nozzle shapes, including open nozzle shapes, were subsequently chosen. Furthermore, mathematical physics analyses were performed to obtain the character of the critical flow, i.e., the course of pressure and its gradients, as a basis for the subsequent evaluation of the dispersion of the primary electron beam passing through the medium. The theory of one-dimensional isentropic flow application assumes significant pressure and temperature gradients [34].

Navier–Stokes equations are used in the Ansys Fluent system for calculations. These equations are partial nonlinear differential equations of the second order. They cover every aspect of real fluid behavior, including turbulence. The finite volume method was used to solve these equations.

The Navier–Stokes equation in a component formulation is as follows:

(7) $$\frac{Du_{i}}{D_{t}}=\frac{\partial u_{i}}{\partial t}+u_{k}\frac{\partial u_{i}}{\partial x_{k}}=-\frac{1}{\varrho }\frac{\partial p}{\partial x_{i}}+\upsilon \frac{\partial^{2}u_{i}}{\partial x_{k}\partial x_{k}}$$

The physical importance of the individual terms of a Navier–Stokes equation is defined as follows:

$\frac{\partial u_{i}}{\partial t}$ is the variability of the flow field over time, $u_{k}\frac{\partial u_{i}}{\partial x_{k}}$ characterizes convection, $-\frac{1}{\varrho }\frac{\partial p}{\partial x_{i}}$ is the pressure gradient, and $\upsilon \frac{\partial^{2}u_{i}}{\partial x_{k}\partial x_{k}}$ is the effect of viscosity.

Then, the transport energy equation is as follows:

(8) $$\frac{\partial \left(\varrho E\right)}{\varrho t}+\nabla \left[V\left(\varrho E+p\right)\right]=\nabla \left[k_{eff}\nabla T-\sum_{j}h_{j}J_{j}+\tau_{eff}V\right]+S_{h}$$

where *k_eff_* is the effective conductivity of the fluid and ***J****_j_* is the diffusion flux of element *j*. The first three terms on the right-hand side of the equation represent energy transfer due to conduction, element diffusion, and viscous dissipation. The physical importance of the individual terms of a transport energy equation is defined as follows:

$\frac{\partial \left(\varrho E\right)}{\varrho t}$ is the change in density and energy over time, $\nabla \left[V\left(\varrho E+p\right)\right]$ is heat transfer through the line (inlet and outlet), $k_{eff}\nabla T$ is heat transfer through the conduction inside the system, $\underset{j}{\sum }h_{j}J_{j}$ is heat transfer by diffusion, $\tau_{eff}V$ is viscous dissipation, and $S_{h}$ is the enthalpy of the system.

Furthermore, energy *E* per mass unit is calculated as follows:

(9) $$E=h-\frac{p}{\varrho }+\frac{V^{2}}{2}$$

where *E* is energy, *T* is time, *ρ* is density, ***V*** is velocity, and *p* is pressure [35].

The results obtained from the theory of one-dimensional isoentropic flow show the occurrence of large pressure gradients, which, at the same time, induce large temperature gradients [35,36]. The Density-Based solver setting was used for this reason. This type of solver solves the governing equations of continuity, momentum, energy, and transport of substances simultaneously as systems of equations. The other governing equations are solved sequentially. They are separated from each other and from a connected set to solve the conjugate set of equations. The solver proved itself to be a more economical choice of implicit formulation of the linearization of the conjugate equations due to the complexity of the flow in the nozzle [37]. In this implicit formulation, each equation in the conjugate set of control equations is linearized implicitly concerning all dependent variables in the set. This formulation further resolves all variables in all cells together. This choice proved to be stable and suitable for the complex case of large pressure gradients in supersonic flow with a significant pressure drop.

The Advection Upstream Splitting Method (AUSM) scheme was chosen as an additional setting. This improved method uses eigenvalues of the Jacobian flow matrices. Convective and pressure flows are formulated using this method. The main features of AUSM, among others, are the accurate capturing of shock and contact discontinuities, entropy-satisfying solutions, uniform accuracy and convergence rate for all Mach numbers, and the lack of “carbuncle” phenomena. The carbuncle phenomenon is a shock instability that appears when numerical low-dissipative shock-capturing techniques are used. This method does not specifically require eigenvectors. For this reason, this method is suitable for systems whose eigenstructure is not explicitly known. These are, for example, systems of two-fluid equations for multiphase flow.

A second-order upwind scheme was chosen to solve the transfer of results between the cellular mesh. Here, the variables on the cell surfaces are calculated using a multivariate linear reconstruction [38,39]. Higher-order accuracy is achieved at cell faces using the Taylor series of solution-targeted expansion around the cell’s center of gravity [40,41] in this approach.

The overall setup of the system was able to cope with the flow rate with all the changes induced during pumping. Furthermore, the setup fully mastered this type of very complex flow and corresponded to the results of experimental measurements. The given mathematical physics analysis required a well-chosen mesh.

**Figure 9:**
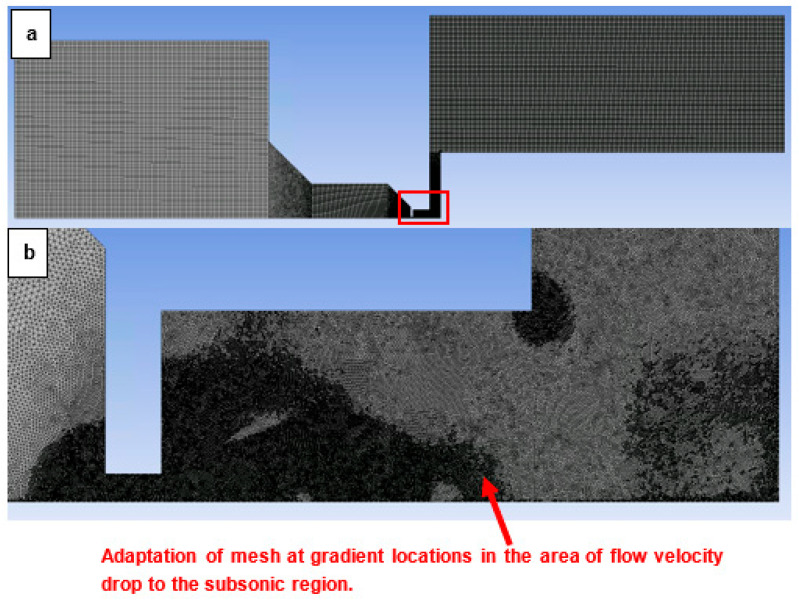
Structured mesh for the mathematical physics analysis (**a**); zoomed area with mesh refinement (**b**).

A combination of a structured mesh with a 2D variant of hexagonal elements was created. These elements are known for their advantage of reduced blurred results caused by a possible error in transferring results over oblique edges. At the same time, they are known for saving the number of cells when meshing purely rectangular surfaces (Figure 9a). In areas where it is not possible to use a high-quality structured mesh, triangular elements were used. It is a narrowed area in the aperture, the nozzle, and behind the nozzle. This area assumes a supersonic flow. In this area, there was a significant refinement of the mesh (Figure 9b) due to the expected large gradients and shock waves [42].

Sensitivity analysis was performed by multiple mesh refinement. Using manual adaptation with the Field Variable register, by gradually increasing the value of the cells above the setting for the Derivative Option Gradient for the static pressure variable, the sensitivity analysis came out negative. The convergent criterion was set to continuity, x-velocity, y-velocity, and energy 1 × 10^3^. At the same time, however, monitoring of the average value of static pressure, velocity, and static temperature in the entire computational volume was performed, and the calculation—even though the convergent criteria of the residues were met—took place until the values of the monitors were equalized. The mesh adjustment range was selected according to the maximum values in the cell’s derivative option, gradient pressure with maximum refinement level 4. As a result, pressure gradients in the areas of supersonic flow in the nozzle were appropriately sensed.

In previous analyses, the tuning of the Low-Pressure Boundary Slip setting [43] was successfully tested. The Slip Flow mode was selected, which was set on the walls in the Ansys Fluent system. This mode respects the lower pressure conditions according to the Maxwell model [38].

(10) $$U_{w}-U_{g}=\left(\frac{2-\alpha_{v}}{\alpha_{v}}\right)K_{n}L_{c}\frac{\partial U}{\partial n}\approx \left(\frac{2-\alpha_{v}}{\alpha_{v}}\right)\frac{\lambda }{\delta }\left(U_{g}-U_{c}\right)$$

(11) $$V_{g}\equiv {\left(\vec{V}\vec{n}\right)}_{g}=V_{w}$$

where *U* and *V* are defined as components of velocity that are parallel and perpendicular to the wall. The indices *g*, *w*, and *c* indicate the gas velocities, the walls, and the center of the cell. *δ* is the distance from the center of the cell to the wall. *L_c_* is the characteristic length. *α_v_* is the adjustment coefficient of the gas mixture, and its value is calculated as the mass-weighted average of each gas in the system.

This variant was chosen for an evaluation of the solution of the given mathematical Maxwell model for ESEM conditions with the expected effects of low slip. Finally, a grid independence study was performed. Manual adaptation of the mesh for the cell in-range version over the entire pressure range was performed again. This adaptation was used for areas with minimal variable changes with the choice of maximum refinement level 2. The area before entering the aperture, the area in the nozzle, and the area of gas expansion used maximum refinement level 4.

In the course of the next calculation, there was no further change in the monitoring in the Ansys Fluent system. Global parameters such as Absolute Pressure, Static Temperature, velocity, and density were monitored. Parametric points were also observed—at selected points of the aperture throat and at five points of 2 mm each in the direction of the gas flow above the aperture. The analysis of mesh independence showed a match and, thus, a sufficiently fine mesh for the given type of analysis.

## 3. Results

**Figure 10:**
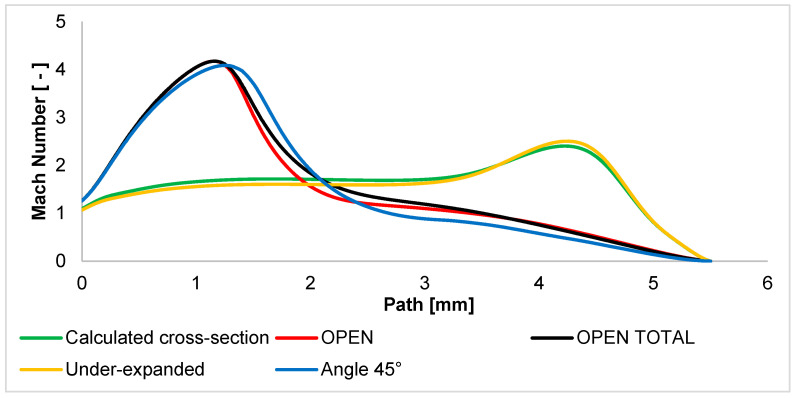
Mach number layout of each variant of the nozzle in the primary electron beam path.

**Figure A1:**
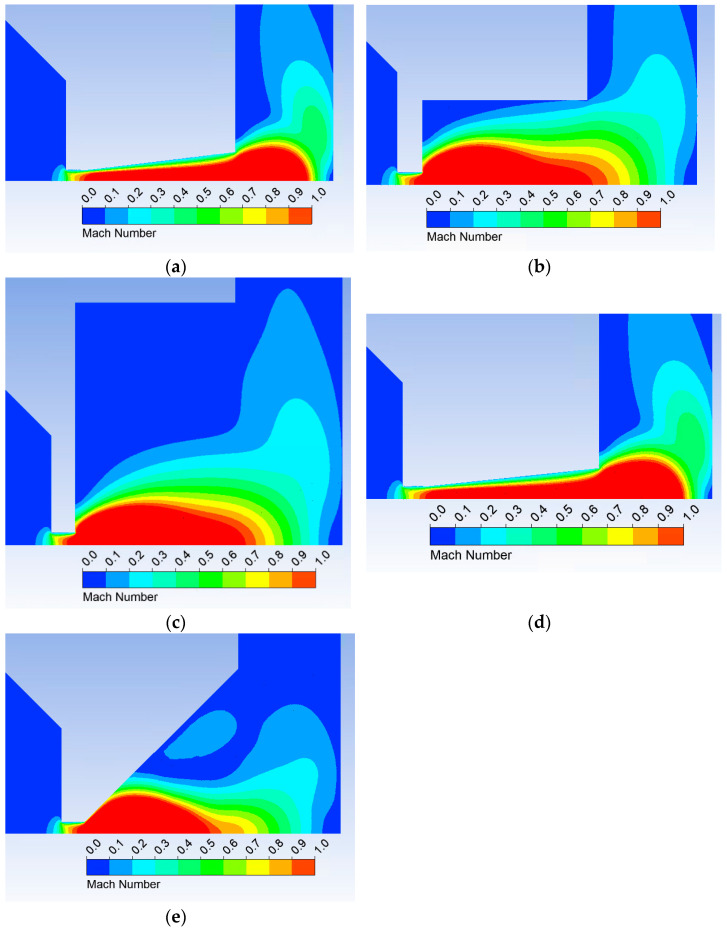
Mach number distribution of calculated cross-section variant (**a**), OPEN variant (**b**), OPEN TOTAL variant (**c**), under-expanded variant (**d**), and angle 45° variant (**e**).

The Mach number is evaluated as a first result. The other evaluated variables are derived from this evaluation. Figure 10 shows the Mach number of each variant. The figure clearly shows different flow patterns between the open variants and the controlled expansion variants. In the calculated cross-section variant, the gas flowing out of the aperture is controlled according to the nature of the expansion given by the pressure ratio between the two chambers. In the graphical layout, this phenomenon can be observed in Appendix A in Figure A1a, where the velocity profile almost copies the shape of the nozzle. Since the controlled expansion calculation states the ideal state, the actual state is changed by the closed environment, which changes the character of the distribution of the *P_v_* back pressure, and thus, there is a slight additional expansion. However, compared to the other variants, this is a controlled expansion.

An under-expanded nozzle behaves very similarly. The difference is that due to the smaller angle in the nozzle area, the gas flow is compressed—under-expanded. For this reason, the Mach number in this area is slightly lower than in the calculated cross-section variant. Behind the nozzle where the gas flow is released, there is a slight additional expansion and increase in the Mach number. This state is evident in Figure A1d, where the velocity profile after the end of the nozzle obtains a more significant extension.

On the other hand, there are variants of the OPEN character. This is where uncontrolled expansion occurs, i.e., an immediate and significant increase in Mach number just behind the aperture. A small difference between the OPEN and OPEN TOTAL variants is that in the OPEN TOTAL variant, the supersonic cone of the flow is not cramped and affected by anything, and there is totally uncontrolled expansion. Figure A1b,c show that at first glance, in the OPEN variant, the region of fast-flowing gas is not affected, but the graphic (Figure 10) shows that at a distance of about 1 mm, the gas flow is slightly cramped, and there is a faster decrease in gas velocity.

It explains the behavior of an over-expanded nozzle with a cone angle of 45°. This nozzle shows that as soon as the nozzle opening angle is greater than the angle of the calculation cross-section variant, uncontrolled expansion occurs. Therefore, the 45° angle variant is much more similar to the OPEN variants, although the shape of the nozzle is more similar to the calculated cross-section variant and the under-expanded variant. The only difference is that the influence and narrowing of the outflowing flow occurs much closer to the aperture. Therefore, the increase in Mach number is slightly braked just behind the aperture. One significant difference with this variant is that the velocity of the supersonic flow drops to the subsonic speed—below 1 Mach—significantly faster, from a distance of about 2.5 mm, while in both OPEN variants, it occurs from up to 1 mm further. In Figure A1e, a large extension of the velocity cone just behind the aperture can be seen. That is, there is very strong expansion in the places where the back pressure causes the gradual termination of the expansion and the compression of the velocity cone back. A graphic distribution of the Mach number of all variants is shown in Appendix A in Figure A1.

**Figure 11:**
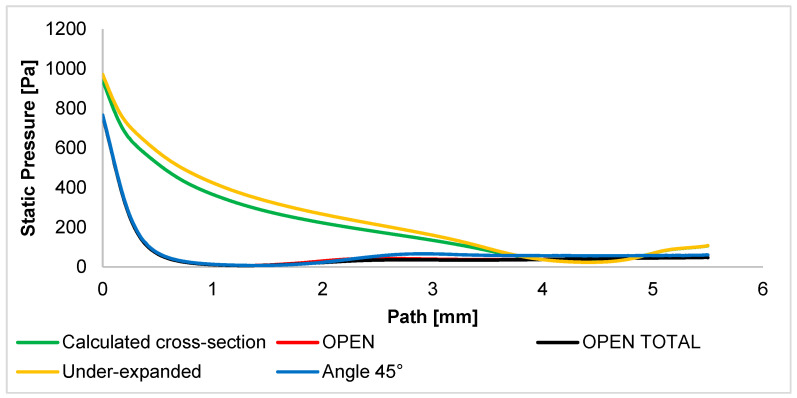
Static pressure layout of each variant of the nozzle in the primary electron beam path.

These Mach number analyses are the key to understanding the course of other variables, especially static pressure. Thus, the static pressure course (Figure 11) is based on the Mach number course. Therefore, these examined variants can also be divided into two groups.

**Figure A2:**
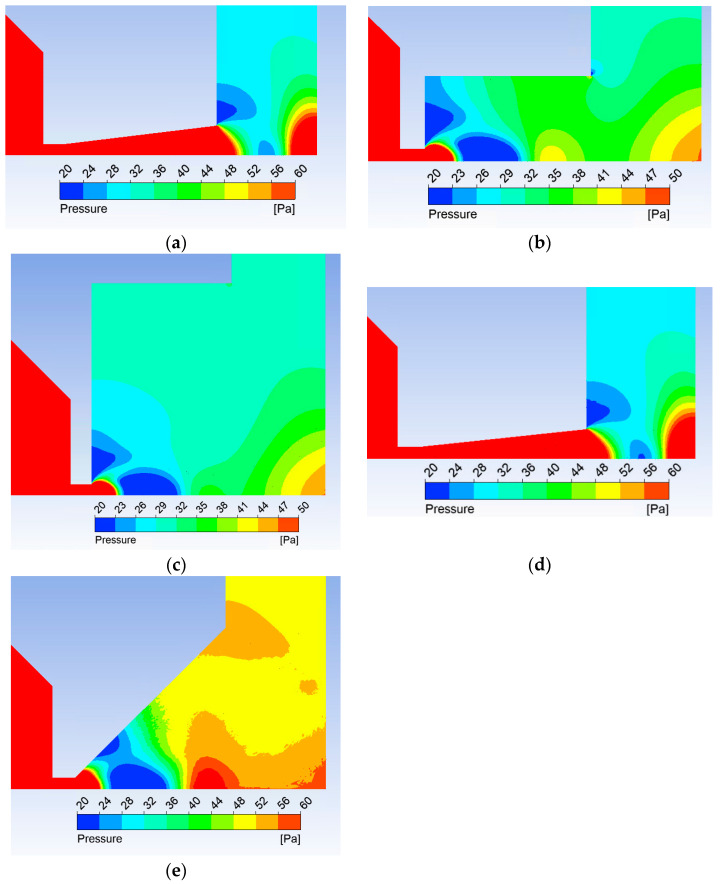
Static pressure distribution of calculated cross-section variant (**a**), OPEN variant (**b**), OPEN TOTAL variant (**c**), under-expanded variant (**d**), and angle 45° variant (**e**).

The first group consists of variants with controlled expansion. The calculated cross-section variant with purely controlled expansion and the under-expanded variant have a static pressure distribution influenced by a slow increase in the Mach number. For this reason, the static pressure decreases significantly more slowly. Figure A2a,d show this fact by a long area of higher pressure, a slight drop in the area of the Mach cone, and a large increase in pressure in the area of sharp gas deceleration in front of the wall.

The second group consists of other variants. In these variants, the static pressure drops sharply due to the sharp increase in the Mach number. Very small differences in the course of the static pressure are given by the previously described small differences in the course of the Mach number from distances of about 1.5 mm. After the end of the supersonic flow, the pressure increases again in the 45° angle variant because, in this variant, the velocity of the flow decreases faster into the subsonic region, which affects the pressure increase, as mentioned earlier. These facts can be observed in detail in Figure A2b,c,e. In these figures, it is possible to observe a sharp drop in pressure very shortly beyond the aperture with a significant Mach disk and its regrowth after the end of the supersonic flow. The graphical distribution of the static pressure of all variants is shown in Appendix A in Figure A2. Static pressure was experimentally sensed using pressure sensors (DPS 300multirange differential pressure sensors for gases and air).

**Figure 12:**
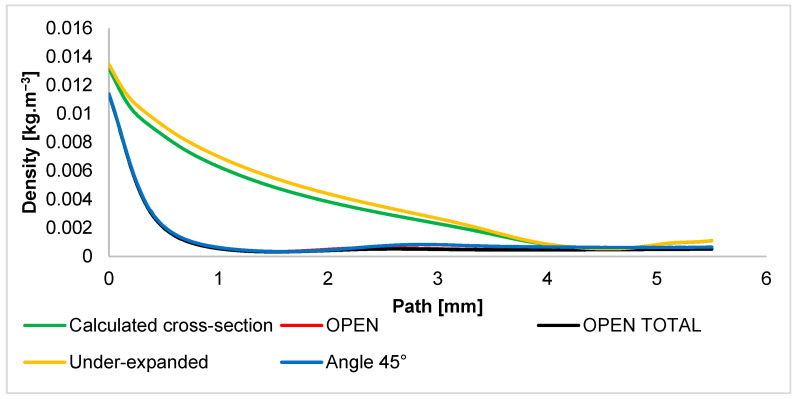
Density layout of each variant of the nozzle in the primary electron beam path.

The distribution of the quantity of density has the same character as the distribution of static pressure. The density course is described in Figure 12, which is shown below.

It is necessary to describe the course of the Static Temperature for a complete flow description. It has a dramatic course due to the supersonic flow. Furthermore, the Static Temperature is strongly dependent on the Mach number course. It is possible to obtain an idea of the Mach number and static pressure curves using experimental measurements of Static Temperature. Static Temperature can be well sensed using temperature sensors (custom-made K-type thermocouple with a diameter of 3 mm). These temperature sensors were chosen because of their ability to operate in cryogenic temperatures. These cryogenic temperatures in the ESEM occur because of supersonic flow behind the aperture.

**Figure 13:**
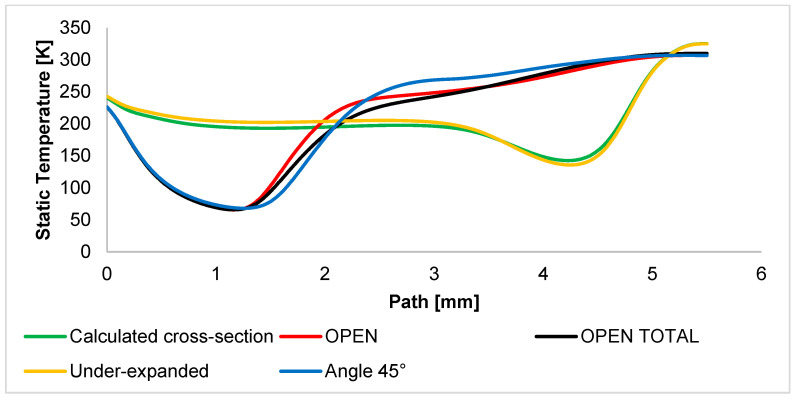
Static Temperature layout of each variant of the nozzle in the primary electron beam path.

**Figure A3:**
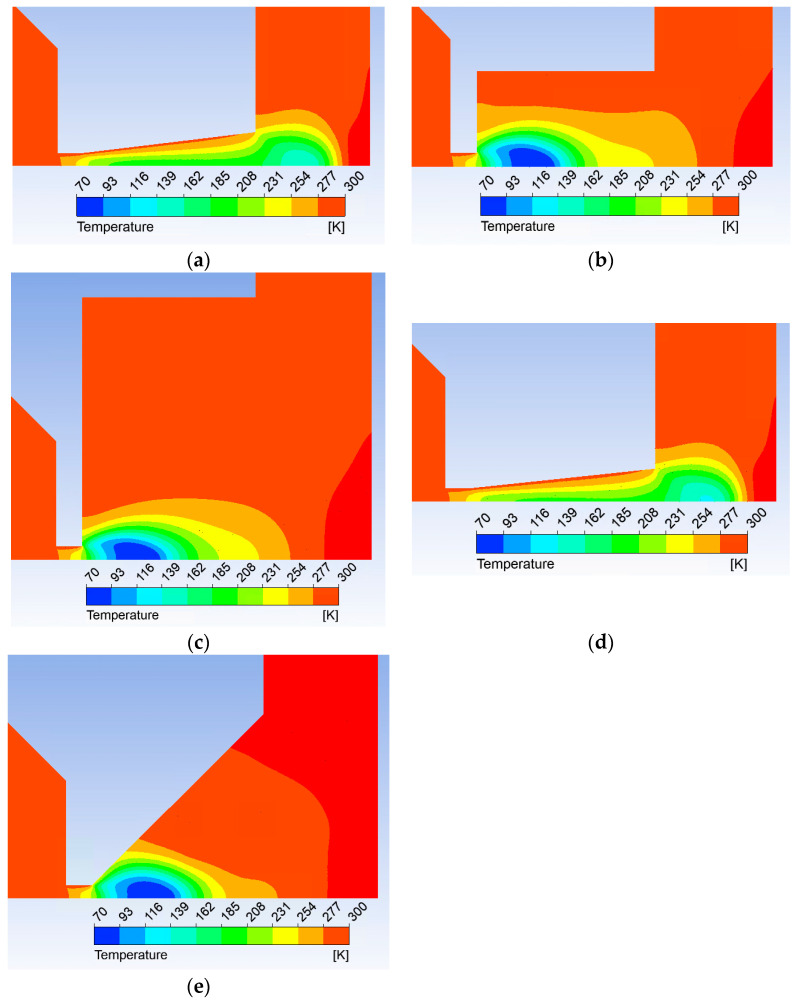
Static Temperature distribution of calculated cross-section variant (**a**), OPEN variant (**b**), OPEN TOTAL variant (**c**), under-expanded variant (**d**), and angle 45° variant (**e**).

The Static Temperature course (Figure 13) is strongly influenced by the supersonic flow course. Therefore, the temperature drops to cryogenic temperatures in regions of supersonic speed. In the OPEN variants, the temperature drops immediately after the aperture, where the gas velocity increases greatly in uncontrolled expansion. In variants with controlled expansion, there is a gradual drop in temperature to the point where the nozzle ends, and additional expansion and velocity increase. From this point, there is a sharp drop in temperature. The overall character of the temperature drop and rise corresponds to the Mach number. For this reason, mapping the temperature course using sensors is very suitable for the experimental part [44]. This behavior is also shown in Figure A3a–d, where the characteristics are analogous to the static pressure waveform. In the above figures, it is possible to observe a drop in temperature in the area of supersonic flow and a sharp drop in pressure up to the mentioned cryogenic temperatures. The graphical distribution of the Static Temperature of all variants is shown in Appendix A in Figure A3.

**Figure 14:**
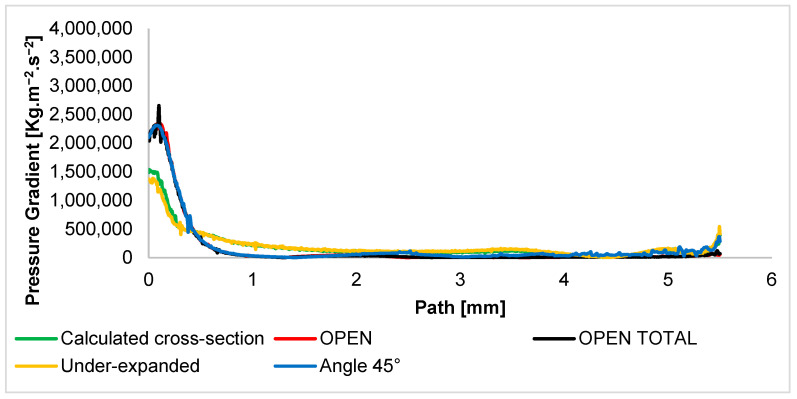
Pressure gradient layout of each variant of the nozzle in the primary electron beam path.

The Mach number course has a major impact on the formation of shock waves. Therefore, for the description of the character of supersonic flow, it is necessary to know the distribution of the pressure gradient (Figure 14). At the same time, the pressure gradient affects the passage of the primary electron beam. This is because each region of increased pressure affects the scattering of the electron beam.

**Figure A4:**
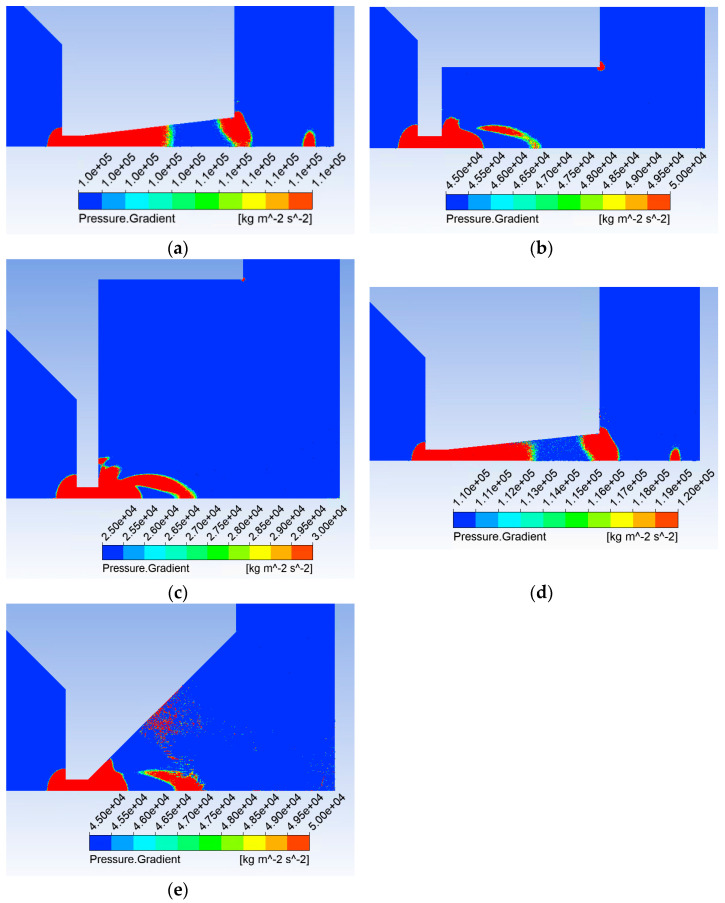
Pressure gradient distribution of calculated cross-section variant (**a**), OPEN variant (**b**), OPEN TOTAL variant (**c**), under-expanded variant (**d**), and angle 45° variant (**e**).

The narrowing in the differentially pumped chamber prevents the formation of classic shock waves with sharp transitions [2], as can be seen in Figure 14. The gas does not flow out from the aperture into free space, but on the contrary, this flow is blocked and slowed down by the wall at a distance of 5.5 mm. It is a braked flow. Therefore, there are no sharp changes in pressure. Thus, the pressure gradients have a slight decline in all variants. The graphical distribution of pressure gradients of all variants is shown in Appendix A in Figure A4, where it is possible to point out the fact that the calculated cross-section and the under-expanded variants cause the formation of two shock waves in a row, but of relatively lower intensity, while the OPEN variants cause only one behind the abruptly terminated region of the supersonic flow.

### Evaluation of Electron Dispersion for Each Variant

The electron beam interacts with the gas molecules in an electron microscope. To determine the collision of one molecule with other molecules of the same species per unit of time *z_A_*, the following can be used:

(12) $$Z_{A}=\pi d^{2}\bar{v}\frac{N}{V}\left[s^{-1}\right]$$

where *d* is the effective collision average.

The number of collisions of one molecule with other molecules of the same species per unit of time is then calculated as follows:

(13) $$z_{A}=\pi d^{2}\bar{v}\sqrt{2}\frac{N}{V}$$

Next, it is necessary to determine the mean free path *l*, which is the average path that a particle travels between two collisions:

(14) $$\bar{l}=\frac{\bar{V}}{z_{A}}$$

From the above, if the number of particles in a unit volume is doubled—i.e., the pressure of the gas—the mean free path drops by half. Furthermore, the mean free path *l* does not depend on temperature [45].

Due to the fact that the scattering of electrons does not depend on temperature, but depends mainly on pressure, it is necessary to investigate the shaping of the aperture and nozzle. This is in order to form shock waves, where there is a significant increase in pressure, in order to eliminate the influence of perpendicular shock waves and limit the influence of oblique shock waves.

The above-mentioned leads to primary electron dispersion. In electron microscopes operating under higher-pressure conditions (ESEM), a portion of the electrons of the primary beam remains in the original path even after passing through the gaseous medium. This portion of electrons creates a signal by interacting with the sample, similar to a conventional electron microscope.

The collision of electrons with atoms and gas molecules occurs by passing the primary electron beam through a medium with higher gas pressures. Electrons can lose some of their energy in these collisions. This can change the direction of their trajectory. If the average number of collisions *M* in a gaseous medium is small, the resulting deviation of the electron from the original beam path is also small. It occurs at the moment when the electron reaches the location of the sample. The length of the electron’s path can then be equal to the thickness of the layer of gas *d* through which the electron is moving. The average number of collisions per electron can be determined from Equation (15) [46].

(15) $$M=\frac{\sigma_{T}Pd}{kT}$$

where *σ_T_* is the total gas gripping cross-section, *P* is the static pressure, *d* is the thickness of the gas layer through which the electron passes, *k* is the Boltzmann constant, and *T* is the absolute temperature.

Then, for the value of *M*, the following apply:

- *M* < 0.05: there is a minimum beam dispersion of up to 5%;
- *M* = 0.05–3: there is partial dispersion in the range of 5–95%;
- *M* > 3: there is complete dispersion above 95%.

**Figure 15:**
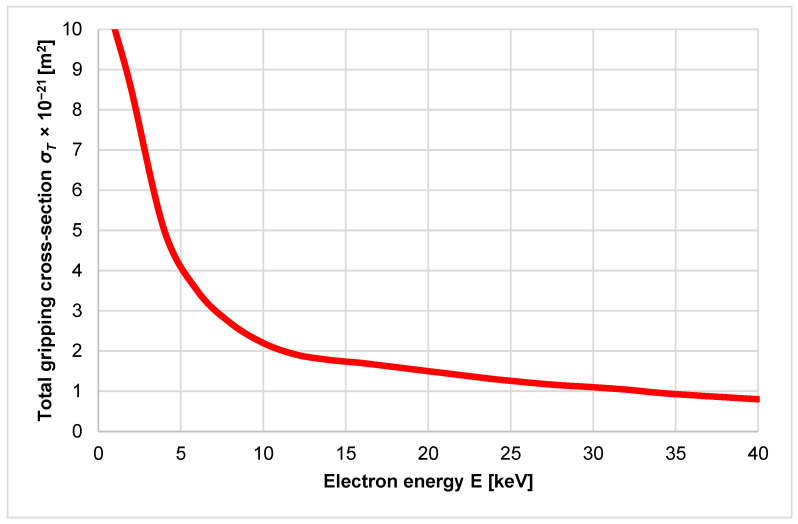
Dependence of gripping cross-section on electron energy.

The gripping cross-section *σ_T_* is defined as the close neighborhood of a gas particle. A collision occurs if the electron finds itself in this cross-section during its passage. The gas gripping cross-section depends on the type of gas and also on the accelerating voltage. The accelerating voltage is defined as the voltage required to form the primary electron beam. The size of the total gripping cross-section of the gas decreases as the energy of the primary electrons increases, as shown in Figure 15. From the point of view of collisions, it is, therefore, advisable to choose the energy of the primary electrons just beyond the bend of this curve so that the flow of primary electrons is scattered as little as possible. Therefore, for mathematical physics analyses, the value of the gripping cross-section was chosen as *σ_T_* = 2 × 10^−21^ [m^2^] for Nitrogen [47].

**Figure 16:**
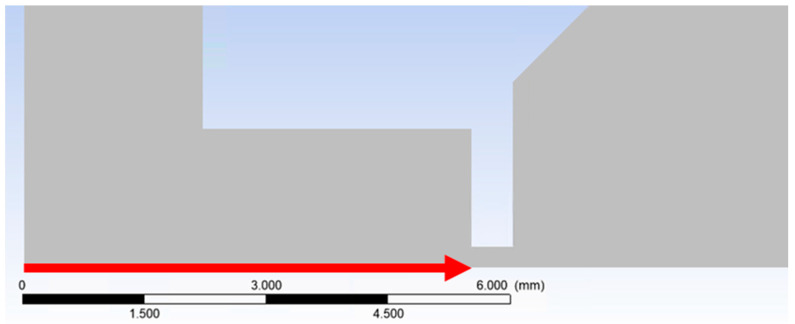
The direction of the primary electron beam path.

Figure 2 shows the path (axis) of the primary electron beam in the ESEM. However, the beam of primary electrons runs against the flow direction, as shown in Figure 16.

**Figure 17:**
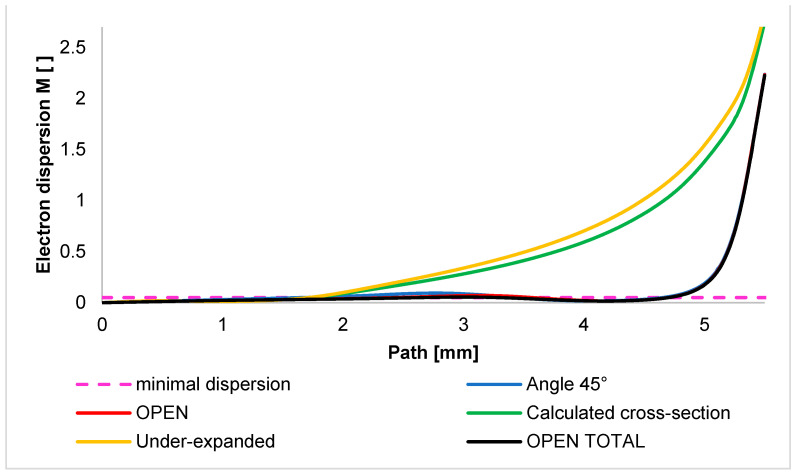
Course of electron dispersion value on the primary electron beam path.

The graph in Figure 17 shows the investigated dispersion path of 5.5 mm passing through a differentially pumped chamber. Thus, the electron dispersion in the braked gas flow is mapped on this path.

Figure 17 shows the course of the electron dispersion value *M* in a simplified way on this path. This describes the magnitude of the dispersion at any given point on this path. It is evident that in the calculated cross-section variant and the under-expanded variant, there is a faster increase in the size of the beam dispersion. In these variants, there is a controlled expansion with a slower increase in velocity and, thus, a slower pressure drop. In contrast to the state of free flow, there are no peaks in the shock waves [2].

**Figure 18:**
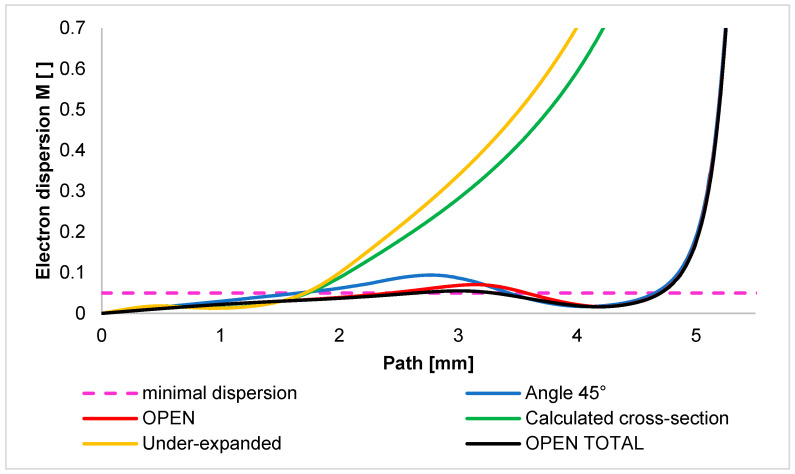
Course of electron dispersion value on the primary electron beam path—adjusted scale range.

Other variants of the OPEN type, including the nozzle with a 45° angle, have a sharply reduced pressure zone immediately behind the aperture due to the high increase in the Mach number. For this reason, almost the entire path of the primary electron beam passing through the differential pumped chamber remains below the minimal dispersion limit. To make it easier to see the differences between the variants, the scale range has been adjusted in Figure 18. This graph shows that the completely uncontrolled expansion of the OPEN TOTAL variant shows a value of the lowest dispersion almost not exceeding the value of the minimal dispersion M = 0.3 throughout the path. On the other hand, the previously described sideways effect leads to the uncontrolled expansion of the OPEN and ANGLE 45° variants, and thus, a slight reduction in the flow velocity results in a slight local increase in the dispersion value.

## 4. Conclusions

A combination of experimental measurements using pressure and temperature sensors in conjunction with the theory of one-dimensional isentropic flow and mathematical physics analyses was presented in this paper. This conjunction serves as a tool for the analysis and subsequent design of nozzles for use in environmental electron microscopy, especially in the differentially pumped chamber. In the beginning, experimental sensing of pressure conditions between the specimen chamber and the differentially pumped chamber was performed. Comparisons were made to determine the usual operating conditions and the ability of vacuum pumps to maintain a given operating pressure ratio for an operating opening, PLA 1, with a diameter of 0.5 mm. From the findings of the experimental measurements, the boundary condition for mathematical physics analyses was determined as the pressure ratio between the chambers (*p*_0_ = 2000 Pa and *p_v_* = 26 Pa). Next, on a fine-tuned mathematical physics model, analyses of the impact of nozzle shaping, located behind the aperture, on the character of the supersonic flow and the subsequent electron beam dispersion were performed. Initially, a calculated cross-section variant of the simplified Laval nozzle was chosen for the investigation, which ensured controlled expansion. Subsequently, an under-expanded nozzle shape was chosen, as opposed to over-expanded variants, where the angle of the linear shape of the Laval nozzle was significantly increased to 45°. To this, we added variants with slightly open space (OPEN variant) and completely open space (OPEN TOTAL variant). The results also showed that nozzle shapes causing an under-expanded gas outflow from the aperture cause a significantly higher electron dispersion value than variants with shapes causing an over-expanded gas outflow. This is due to the control of the flow velocity and, thus, the controlled reduction in the static pressure curve on the path of the primary electron beam. The flow character in the aperture leading into a limited space and its impact on electron dispersion for the purposes of ESEM research were also investigated in this paper. These results will be used for further research in the field of differentially pumped chambers.

## Figures and Tables

**Table 1:** Specifications of pressure sensors Pfeiffer CMR 361 and Pfeiffer CMR 362.

	Pfeiffer CMR 361	Pfeiffer CMR 362
Measuring range [Pa]	10–11,000	10–110
Precision: % of measurement [%]	0.2	0.2
Pressure max. [hPa]	3000	2000
Response time [ms]	30	30

**Table 2:** Experimentally measured pressure conditions in the specimen chamber and the differentially pumped chamber.

Pressure [Pa]	
Specimen Chamber	Differentially Pumped Chamber
*p*_0_	*p_v_*
50	1.25
100	2.20
200	3.80
300	5.20
400	6.70
500	8.10
600	9.40
700	10.80
800	12.10
900	13.40
1000	14.70
1100	15.90
1200	17.10
1300	18.30
1400	19.50
1500	20.75
1600	22.10
1700	23.20
1800	24.30
1900	25.20
2000	26.00

## Data Availability

The data presented in this study are available on request from the corresponding author.
